# Effects of Bait on Male White-Tailed Deer Resource Selection

**DOI:** 10.3390/ani11082334

**Published:** 2021-08-07

**Authors:** James T. Johnson, Richard B. Chandler, L. Mike Conner, Michael J. Cherry, Charlie H. Killmaster, Kristina L. Johannsen, Karl V. Miller

**Affiliations:** 1 D.B. Warnell School of Forestry and Natural Resources, The University of Georgia, 180 E. Green Street, Athens, GA 30602, USA; rchandler@warnell.uga.edu (R.B.C.); kmiller@warnell.uga.edu (K.V.M.); 2 The Jones Center at Ichauway, 3988 Jones Center Drive, Newton, GA 39870, USA; mike.conner@jonesctr.org; 3 Caesar Kleberg Wildlife Research Institute, Texas A&M University-Kingsville, 700 University Blvd, MSC 218, Kingsville, TX 78363, USA; michael.cherry@tamuk.edu; 4 Georgia Department of Natural Resources, Wildlife Resources Division, 2067 US Highway 278 SE, Social Circle, GA 30025, USA; charlie.killmaster@dnr.ga.gov (C.H.K.); tina.johannsen@dnr.ga.gov (K.L.J.)

**Keywords:** camera survey, Georgia, *Odocoileus virginianus*, space-use, spatial capture-recapture

## Abstract

**Simple Summary:**

Bait is often used to attract wildlife to enhance viewing opportunities, increase harvest rates, or to improve population survey methods for research and management purposes. However, baiting wildlife can alter animal behavior, leading to negative outcomes such as increased disease transmission, competition, and susceptibility to predation. Our objectives were to determine the effects of short-term baiting on male white-tailed deer behavior and distributions within several properties in southwestern Georgia, USA. We used cameras at baited and unbaited locations to assess the impacts of bait on deer space use within home ranges and to determine whether bait caused shifts in the distribution of home ranges during summer and winter surveys. We found little evidence that short-term baiting affected the distributions of home ranges on the landscape; however, we found evidence that space use within home ranges was affected by bait. By concentrating deer space use within seasonal home ranges, bait may enhance disease transmission and change harvest susceptibility.

**Abstract:**

Bait is often used to increase wildlife harvest susceptibility, enhance viewing opportunities, and survey wildlife populations. The effects of baiting depend on how bait influences space use and resource selection at multiple spatial scales. Although telemetry studies allow for inferences about resource selection within home ranges (third-order selection), they provide limited information about spatial variation in density, which is the result of second-order selection. Recent advances in spatial capture-recapture (SCR) techniques allow exploration of second- and third-order selection simultaneously using non-invasive methods such as camera traps. Our objectives were to describe how short-term baiting affects white-tailed deer (*Odocoileus virginianus*) behavior and distribution. We fit SCR models to camera data from baited and unbaited locations in southwestern Georgia to assess the effects of short-term baiting on second- and third-order selection of deer during summer and winter surveys. We found little evidence of second-order selection during late summer or early winter surveys when camera surveys using bait are typically conducted. However, we found evidence for third-order selection, indicating that resource selection within home ranges is affected. Concentrations in space use resulting from baiting may enhance disease transmission, change harvest susceptibility, and potentially bias the outcome of camera surveys using bait.

## 1. Introduction

Baiting is frequently used to alter behavior of wildlife for desirable outcomes such as increasing harvest success, facilitating population control [1,2,3,4], enhancing wildlife viewing opportunities, increasing detection rates for camera surveys [5,6], and improving capture rates in research efforts [7,8]. However, baiting wildlife can produce undesirable outcomes such as trophic cascades [9], inter- and intra-specific competition [10,11], and increased risk of disease transmission [12,13,14,15]. Many of the negative effects associated with baiting result from altered movement patterns [16,17,18,19] which concentrate wildlife at higher densities. Baiting can congregate species such as white-tailed deer (*Odocoileus virginianus*) in closer proximity than would occur under natural foraging conditions, increasing the chance of horizontal transmission of diseases such as chronic wasting disease (CWD) and bovine tuberculosis through deer-deer contact [12,13,18], as well as indirect contact from contaminated bait sites [20,21]. Baiting deer for hunting purposes is legal to some extent in 26 of the 48 contiguous states in the United States [22]. However, Best Management Practices for management of CWD include elimination of ‘baiting and feeding of all wild cervids using regulatory mechanisms such as jurisdictional bans’ [23].

Several studies have concluded that baiting has little effect on the home range size of white-tailed deer [19,24,25,26], but baiting can affect within home range space use and resource selection [19,26,27]. These studies used various methods (telemetry, human observers, and cameras) to assess the effects of bait on resource selection but focused on a single spatial scale. Resource selection, however, is a hierarchical process [28], often involving the selection of home ranges within a landscape (second-order), and the (third-order) selection of resources within a home range.

Spatially explicit capture-recapture models (hereafter: SCR) allow for simultaneous inference about second- and third-order selection [29,30,31,32]. In the SCR framework, second-order selection is assessed by modeling spatial variation in animal density. If density is uniform across a landscape, then the distribution of home ranges is not the result of selection. Conversely, departures from spatial uniformity in density provide evidence of second-order selection. Third-order selection is assessed by modeling encounter probability as a function of trap-specific covariates while accounting for the distance between home range centers and trap locations [31]. If animals are not exhibiting resource selection within home ranges, encounter rates at traps should not be affected by trap-level covariates.

We assessed the impacts of bait on second- and third-order selection of white-tailed deer to evaluate how bait may influence deer behavior and distribution. In addition, we evaluated how the effects of bait on deer behavior change seasonally, given resource selection is known to vary throughout the year.

## 2. Materials and Methods

### 2.1. Study Area

**Figure 1:**
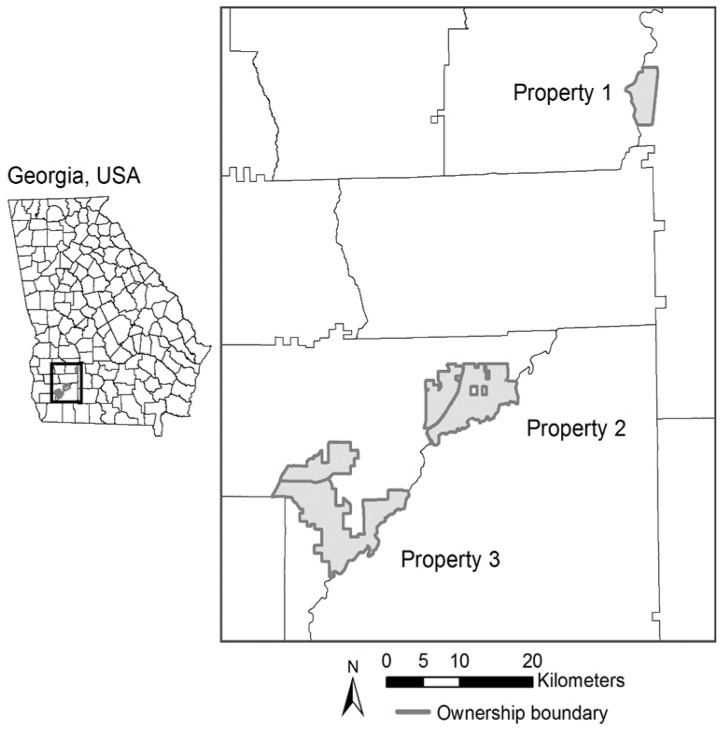
Properties in southwestern Georgia, USA, used to evaluate the effects of bait on male white-tailed deer (*Odocoileus virginianus*) resource selection in 2015–2016. Property one contains camera array site A, property two contains site B, and property three contains sites C and D.

Our study area included four study sites at three privately owned properties in southwestern Georgia, USA, in Worth (31.5282° N, 83.8897° W) and Baker counties (31.2816° N, 84.4803° W) (Figure 1). The properties ranged in size from approximately 1600 to 12,000 ha with varying deer densities and management regimes. Habitat types consisted of longleaf pine (*Pinus palustris*) savannas, scattered hardwoods (primarily oaks; *Quercus* spp.), riparian zones, planted loblolly pine (*P. taeda*) stands, wildlife openings, and depressional wetlands. Typical landscape management for all three properties included frequent prescribed fire (~two-year intervals), wildlife food plots, predator trapping, timber management, roller chopping, mowing, and seasonal disking. Unlike properties two and three, property one had a long-term deer supplemental feeding program.

We conducted camera surveys in late summer (August–September) of 2015 and early winter (January–February) of 2016, which included cameras at both baited locations (hereafter: baited cameras) and unbaited locations (hereafter: passive cameras). The concurrent use of baited and passive cameras gave deer the choice of baited or unbaited locations within their home range, allowing us to quantify the effects of bait on resource selection. We used unique antler configurations to manually identify males and SCR models to investigate the seasonal effects of baiting on second- and third-order selection. We were not able to distinguish female deer to create individual capture histories, and we, therefore, excluded females from the analysis. We established four 1000 ha camera trapping sites within the three properties. Property one contained site A, property two contained site B, and property three contained sites C and D. Within sites B–D, 49 passive cameras (one camera per approximately 20 ha) and 25 baited cameras (one camera per approximately 40 ha) were distributed using systematic random sampling on a grid. Property one had a long history (>10 years) of baited camera use. Therefore, we used the pre-established 29 baited camera locations for this property (one camera per approximately 50 ha) and placed 49 passive cameras (one camera per approximately 20 ha) within the baited camera array. Baited cameras were operated and distributed according to the methodology commonly associated with a camera survey using bait [5]. Unlike the other properties, the majority of baited camera locations on property one were associated with long-term tripod gravity feeders, containing corn or protein pellet supplements; however, feeders remained empty while the baited camera surveys were conducted.

We secured baited cameras on trees ~1.5 m from ground level near the center of each grid cell and placed shelled corn approximately 5 m from each baited camera. Baited cameras were operated for two weeks after a one-wk pre-baiting period [5]. We placed the passive cameras within 200 m of the centroid of each grid cell, on trees or metal fence posts at the same height as baited cameras. To place passive cameras, we searched a 200-m buffer surrounding the centroid of each cell for the highest level of deer activity, such as deer trails and movement corridors, to increase the chances of capturing images of deer.

Both baited and passive cameras were operated simultaneously for two weeks in August-September 2015 (late summer), prior to the onset of deer hunting season, and again for two weeks in January-February 2016 (early winter), immediately following the conclusion of the 2015–2016 Georgia deer hunting season, which is typical for this region when surveying white-tailed deer using bait following the Jacobson et al. (1997) camera survey protocol. All cameras were operated for 24 h d^−1^ during the study period. We checked passive cameras once during the two-week period and visited the baited cameras twice per week to replenish bait if needed. We used the infrared camera model Uway VH200HD (HCO, Duluth, GA, USA).

### 2.2. Spatial Model

**Figure 2:**
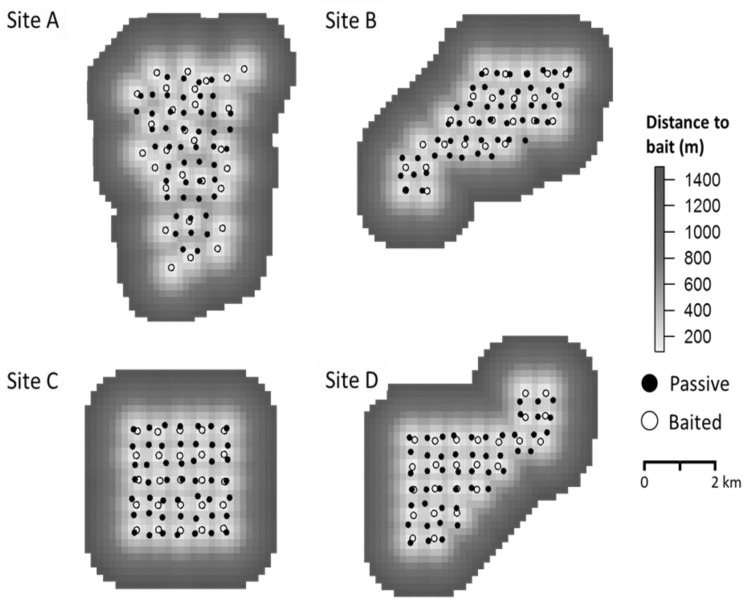
Raster density surfaces of each site (**A**–**D**) located in southwestern Georgia, USA, indicating distance from the spatial covariate, bait. Cameras were operated in the summer of 2015 and winter of 2016 to evaluate the effects of bait on male white-tailed deer (*Odocoileus virginianus*) resource selection. Open circles represent baited cameras and closed circles represent passive cameras.

We used unique antler characteristics of males detected by cameras to identify individuals and create capture histories. We defined each occasion as a 24-h period. We generated a spatial raster layer representing distance to bait (Figure 2). Each raster cell measured 180 m × 180 m and the extent was defined by a 1.5-km buffer surrounding each camera array. We chose the 1.5-km buffer to define a state-space for each site, such that the probability of detecting an individual near the border of the region was negligible [31]. The areas of the four buffered regions at sites A–D were: 41.21 km^2^, 34.83 km^2^, 29.58 km^2^, and 34.15 km^2^, respectively. Other extraneous factors (e.g., habitat variables) which may influence deer distribution and space use were treated as random sources of variation, which SCR models are robust at ignoring.

To assess second-order selection, we modeled spatial variation in density using distance to bait as a spatial covariate. In SCR models, density is characterized by a spatial point process for the activity centers of the N individuals in the population [29,30,31,33]. An activity center is the average location of an individual during the sampling period. For deer with stationary symmetric home ranges, the activity center is the home range center. Therefore, if the density of activity centers is uniform across the landscape, then the distribution of deer home ranges during our study is not the result of selection. We modeled density of activity centers at location s with a log-linear function:

$$\mu \left(s\right)=exp\left(\beta_{0}+\beta_{1}DISTBAIT\left(s\right)\times w\right)\times pixelArea$$

The effect of bait ($\beta_{1}$) can be interpreted in the same way as in generalized linear models [31]. We assumed that $\beta_{1}$ would be less than zero, indicating deer density decreases as distance to bait increases. We did not assess the unrealistic scenario of positive correlation between distance to bait and density. In addition to estimating the effect of bait, we computed the probability that bait had no effect by including an indicator variable w, which would equal one if the data strongly suggest that a bait effect is present. We used a w∼Bern(0.5) prior distribution. The effect of bait was considered significant if Pr(w=0)<0.05, similar to the frequentist *p* value approach.

We used a Bernoulli observation model for the encounter histories, and we used the standard half-normal model for the encounter function:

$$p_{ij}=p_{0}exp\left(\frac{-d_{ij}^{2}}{2\sigma^{2}}\right)$$

where $p_{0}$ is the encounter probability when the distance ($d_{ij}$) between an activity center and a camera trap is zero. We estimated separate $p_{0}$ parameters for baited and passive cameras to determine if bait affected third-order selection. If deer are not exhibiting resource selection within home ranges, encounter rates should not differ between baited and passive traps. Detection probability was set to zero on occasions for which a camera was not operational due to camera malfunctions. We assumed that $p_{0}$ would be greater at baited cameras than passive cameras because deer actively select sites with bait within their home ranges. The parameter σ is the spatial scale parameter describing how encounter probability decreases with increasing distance from a camera site and a deer’s home range center. The scale parameter is proportional to home range size because deer with larger home ranges can be detected farther from their home range centers than deer with smaller home ranges. Spatial capture-recapture models do not assume statistical independence among camera locations. In fact, spatial autocorrelation is useful for estimating σ because it results from recaptures of individuals at multiple locations [31].

We used data augmentation and a Bayesian approach for statistical inference [31]. We used vague prior distributions for all parameters (Appendix A). We fit the model in R [34] with the package rjags [35], which interfaces with the Gibbs sampler software JAGS [36]. We generated two Markov chains each representing 30,000 posterior samples, and we discarded the first 1000 as burn-in. Convergence was graphically assessed and evaluated using the Gelman-Rubin statistic [37].

## 3. Results

We collected 19,904 photographs of 470 uniquely identified males during the two-week concurrent summer survey, and 20,019 photographs of 423 unique males during the two-week concurrent winter survey. Baited cameras produced 40 and 50 times more images of antlered males than passive cameras during the summer and winter periods, respectively (Table 1).

**Figure 3:**
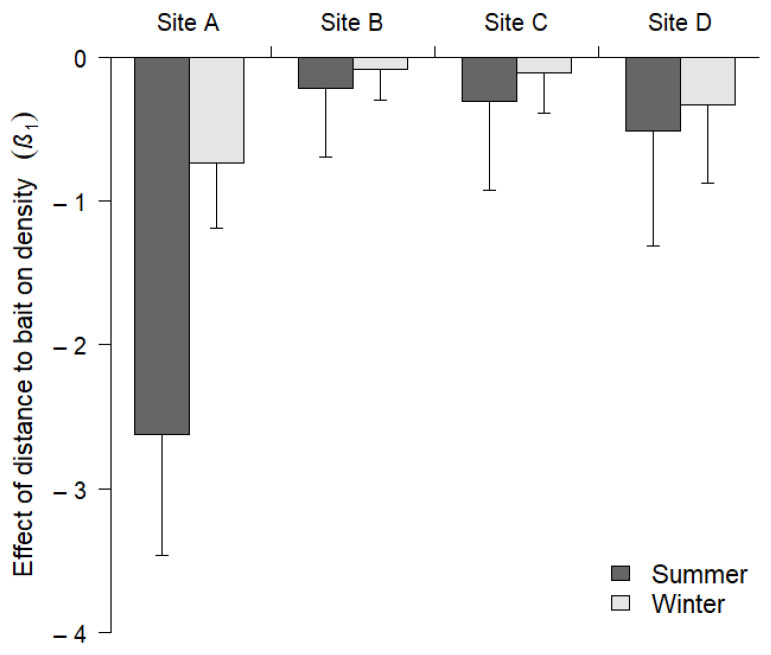
The effect of distance to bait on adult male white-tailed deer (*Odocoileus virginianus*) density distributions within four sites during summer 2015 and winter 2016 surveys in southwestern Georgia, USA.

**Figure 4:**
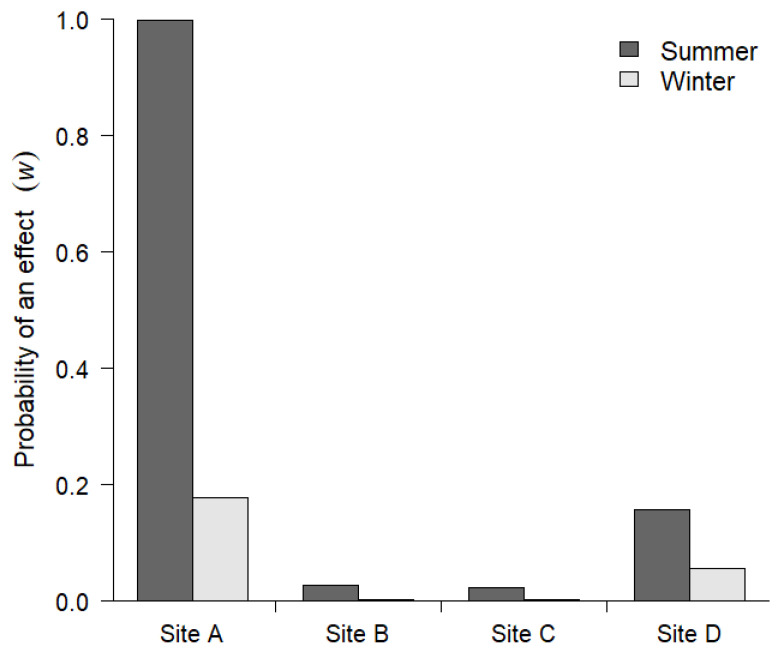
The probability that bait influenced second-order selection of male white-tailed deer (*Odocoileus virginianus*) in southwestern Georgia, USA, during summer (2015) and winter (2016) camera surveys.

We found little evidence that baiting affected second-order selection of male white-tailed deer (Figure 3 and Figure 4). There was no effect of distance to bait on spatial variation in density, *Pr*(*w* = 1) < 0.05, except for site A, and the effect was only evident during the summer survey. The effect size at this site was more than three times greater than any of the other effects (Figure 3). Overall, the effect of bait on second-order selection was weaker during the winter than during the summer (Figure 3 and Figure 4).

**Figure 5:**
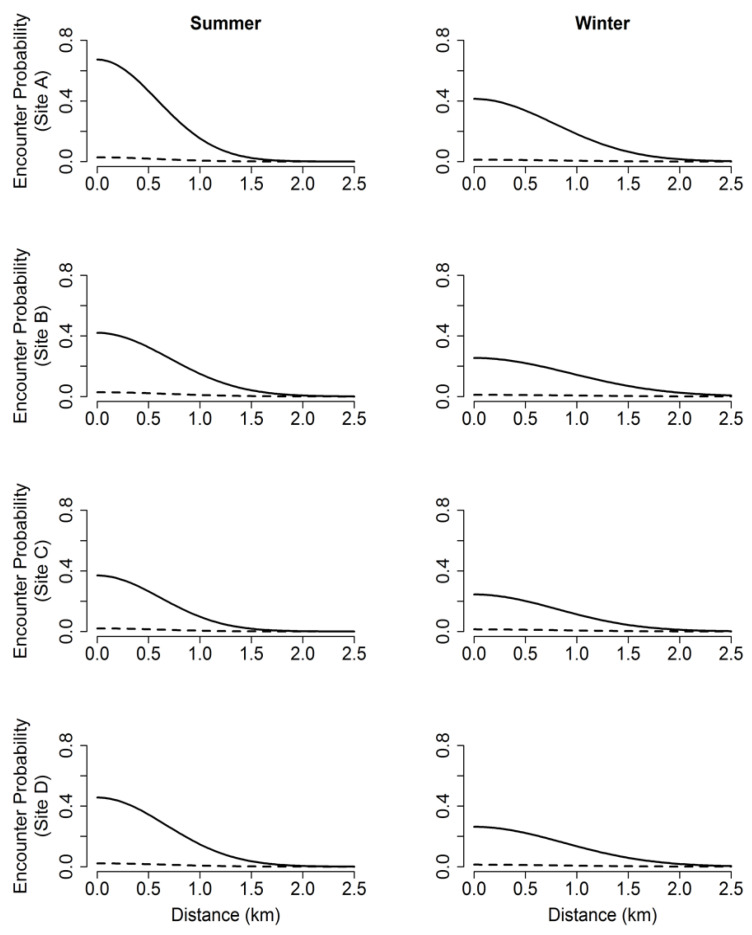
Half-normal detection functions of each baited (solid lines) and passive (dashed lines) camera array site, by season (summer 2015 and winter 2016), describing how the encounter probability of white-tailed deer (*Odocoileus virginianus*) changes as a function of distance (km) from camera at study sites in southwestern Georgia, USA.

**Figure 6:**
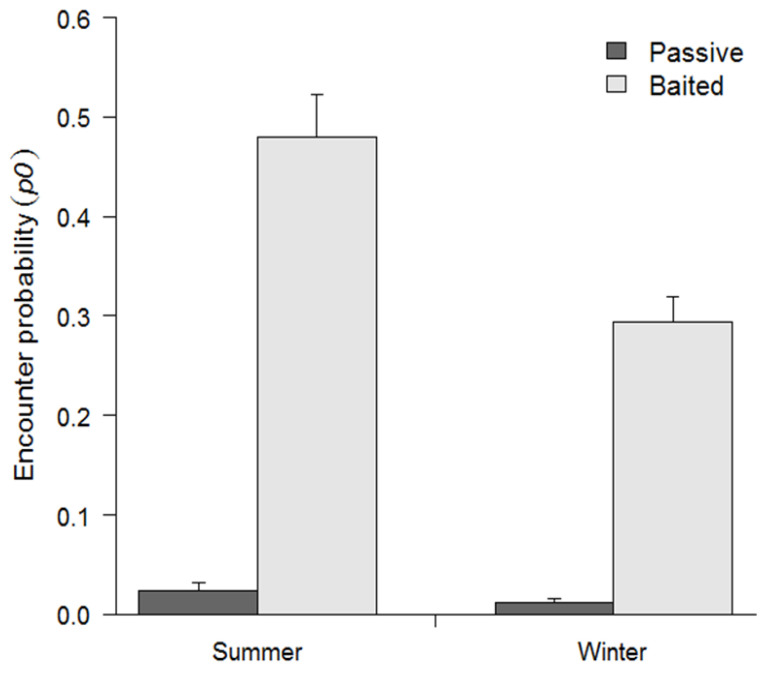
Encounter probabilities (95% CI) of male white-tailed deer (*Odocoileus virginianus*) captured on passive and baited cameras during summer 2015 and winter 2016 surveys at four sites in southwestern Georgia, USA.

We found evidence for third-order selection and baseline encounter probability ($p_{0}$) varied among sites and between seasons (Figure 5 and Table 2). Baseline encounter probability was greater at baited sites than at unbaited sites during both survey seasons and was higher in summer than during winter for both baited and passive cameras. Male deer were 19.6 times more likely to be encountered at a baited site within their home range than passive sites during the summer survey and 23.5 times more likely to be encountered at baited sites than passive sites during the winter surveys. In addition, males were 1.6 times more likely to be encountered at baited sites during the summer surveys when compared to winter surveys, and twice as likely to be encountered at passive sites during the summer when compared to passive sites in the winter (Figure 6).

The spatial scale parameter (σ) was smallest in the summer (0.41 km) and nearly doubled in the winter to 0.720 km, suggesting an expansion in home range size during the winter surveys at our study sites (Table 2). Although it was not a primary objective of our study, the SCR analysis also produced estimates of N. For all sites, we found a decrease in abundance of antlered males after the hunting season, as expected (Table 2).

## 4. Discussion

In general, baiting had little influence on spatial variation in male deer density (second-order selection), suggesting that the distribution of male deer home range centers is not impacted by the presence of short-term baited sites. The only evidence of a relationship between male deer spatial distribution (second-order selection) and baited cameras occurred at site A within the property with a long history of deer management, including spring and summer supplemental feeding programs. However, this association was only apparent during the summer survey of this site, and the effect was significantly reduced during the winter survey after intensive feeding programs were stopped four months earlier. Although we could not address the possibility of long-term shifts in home range selection, our finding that bait exerted limited short-term effects on second-order selection is important from a management perspective because it suggests that bait will not alter landscape-scale distribution during the hunting season. Altered spatial distributions of home ranges can occur when abundant resources, such as agricultural crops, are available for prolonged periods [38,39]. Future research should explore how long bait needs to be present on the landscape for changes in second-order selection to occur.

We did find evidence of third-order selection, with deer selecting baited camera locations within their home ranges at a higher rate than passive camera locations. Our finding of evidence for third-order selection is consistent with previous studies [19,26]. Beaver [26] reported that bait affected core area use by radio-instrumented male deer more than habitat variables such as canopy. Similarly, Kilpatrick and Stober [19] found that radio-instrumented deer did not alter spatial distributions of home ranges after bait was applied but did alter core area use within established home ranges. The framework used in our study alleviates the need to use expensive telemetry-based methods, which do not afford the opportunity of quantifying the effect of bait on spatial variation in density.

Our findings of an effect of bait on third-order selection support previous work indicating that baiting can increase deer-to-deer contact rates [18]. Increased contact rates could ultimately lead to higher transmission rates of diseases in susceptible areas [40,41]. Direct contact between deer is not uncommon in natural settings where bait sites are not present; however, these behaviors are typically associated with small social groups [42,43]. Short-term baited surveys, such as the Jacobson et al. [5] survey, also require continual bait replenishment over time at a single location where a camera is present, likely exacerbating contamination levels [18].

In addition to providing assessments of both second- and third-order selection, SCR models yield estimates of home range size as well as abundance, which is a primary objective of many camera surveys. At all sites, the spatial scale parameter (σ) associated with home range size was approximately two times larger in the winter than in the summer, consistent with the studies reviewed by Marchinton and Hirth [43]. From their work in agricultural landscapes, Nixon et al. [29] and Brinkman et al. [44] reported that deer home ranges were more than twice as large in the winter than in summer. In our study area in southwestern Georgia, reduced home range size during the summer is likely the result of greater cover and forage than in the winter.

Baiting has become a contentious issue in deer management as it has the potential to impact behavior, harvest susceptibility and disease transfer. Although there are many mechanisms by which bait may influence deer populations, most research has focused on the effects of bait on selection at a single spatial scale. Our work presents a framework for assessing the influence of bait on multiple spatial scales, and we have demonstrated that the effects may differ between spatial scales.

## 5. Conclusions

Our results indicate that short-term use of bait (<three weeks) does not strongly affect spatial variation in density, but it does cause deer to concentrate space use near bait sites within home ranges. To our knowledge, this is the first attempt to simultaneously assess the effects of bait on multiple orders of selection within a deer population, and it has important implications for assessing the impacts of bait on deer ecology. Future investigations should seek to understand the long-term effects of baiting, possibly by using before-after-control-intervention experiments over several years. Additional work is also needed to assess multi-scale impacts on does and fawns, which cannot be uniquely identified with camera data.

## Figures and Tables

**Table 1:** Numbers of images and identified male white-tailed deer. Total number of male white-tailed deer (*Odocoileus virginianus*) images collected and total number of unique male deer identified during summer (2015) and winter (2016) baited and passive camera surveys in southwestern Georgia, USA.

	Site A	Site B	Site C	Site D
Summer baited photos	10,613	3609	3006	2188
Summer passive photos	240	143	57	48
Summer unique male individuals	208	93	94	75
Winter baited photos	11,318	3587	2222	2502
Winter passive photos	141	114	67	68
Winter unique male individuals	177	85	89	72

**Table 2:** Male white-tailed deer spatial-capture recapture parameter estimates. Estimates of the posterior means for male white-tailed deer (Odocoileus virginianus) abundance (*N*), intensity function intercept (*β*_0_), the effect of bait on deer activity center density (*β*_1_), baseline encounter probabilities for both passive (*p*_0_ [passive]) and baited cameras (*p*_0_ [baited]), and the scaling parameter of the half-normal detection function (*σ*) from summer (2015) and winter (2016) camera surveys in southwestern Georgia, USA.

			Summer				Winter		
Site	Parameter	Mean	SD	2.5%	97.5%	Mean	SD	2.5%	97.5%
A	*N*	261.5	8.925	247.0	282.0	214.0	5.167	205.0	225.0
	*β*_0_	3.247	0.140	2.972	3.521	2.133	0.134	1.869	2.392
	*β*_1_	−2.621	0.416	−3.456	−1.838	−0.734	0.223	−1.182	−0.309
	*p*_0_ [passive]	0.028	0.002	0.023	0.033	0.012	0.001	0.010	0.015
	*p*_0_ [baited]	0.673	0.017	0.641	0.707	0.414	0.011	0.393	0.436
	*σ*	0.338	0.004	0.331	0.345	0.607	0.008	0.592	0.623
B	*N*	138.6	8.041	124.0	155.0	100.3	1.180	99.0	103.0
	*β*_0_	1.529	0.125	1.312	1.805	1.112	0.099	0.922	1.315
	*β*_1_	−0.210	0.174	−0.651	−0.007	−0.081	0.074	−0.275	−0.002
	*p*_0_ [passive]	0.028	0.004	0.022	0.036	0.011	0.001	0.009	0.014
	*p*_0_ [baited]	0.420	0.023	0.378	0.467	0.255	0.011	0.233	0.278
	*σ*	0.487	0.012	0.463	0.510	0.872	0.018	0.838	0.907
C	*N*	168.5	14.598	141.0	198.0	108.2	4.387	101.0	118.0
	*β*_0_	1.940	0.124	1.719	2.207	1.367	0.108	1.160	1.589
	*β*_1_	−0.302	0.237	−0.874	−0.011	−0.108	0.097	−0.358	−0.003
	*p*_0_ [passive]	0.020	0.003	0.014	0.027	0.014	0.002	0.010	0.018
	*p*_0_ [baited]	0.370	0.021	0.331	0.411	0.244	0.014	0.219	0.272
	*σ*	0.375	0.009	0.358	0.394	0.652	0.019	0.615	0.691
D	*N*	104.6	8.606	89.0	123.0	85.5	2.349	82.0	91.0
	*β*_0_	1.459	0.183	1.137	1.848	1.141	0.174	0.832	1.511
	*β*_1_	−0.509	0.333	−1.263	−0.031	−0.327	0.223	−0.838	−0.016
	*p*_0_ [passive]	0.022	0.004	0.015	0.030	0.013	0.002	0.009	0.018
	*p*_0_ [baited]	0.457	0.025	0.409	0.506	0.263	0.013	0.239	0.289
	*σ*	0.443	0.012	0.421	0.467	0.750	0.019	0.713	0.789

## Data Availability

Ga-deer-resource-selection. Available online: https://github.com/JTJohnsonUGA/ga-deer-resource-selection (accessed on 5 August 2021).

## Appendix A. SCR JAGS Model

model {
p0[1] ~ dbeta(1,1)
p0[2] ~ dbeta(1,1)
sigma ~ dgamma(1,1)
beta0 ~ dnorm(0, 0.1)
beta1 ~ dunif(−100,0)
w ~ dbern(0.5)
for(g in 1:nPixels) {
mu[g] <- exp(beta0 + beta1*bait.dist[g]*w)*pixelArea
pi[g] <- mu[g]/sum(mu)}
EN <- sum(mu)
psi <- EN animals-11-02334M
for(i in 1:M) {
z[i] ~ dbern(psi)
s[i] ~ dcat(pi[])
for(j in 1:J) {
p[i,j] <- p0[bait[j]]*exp(-1*pow(d[s[i],j],2)/(2*sigma^2))
for(k in 1:K) {
y[i,j,k] ~ dbern(p[i,j]*z[i]*oper[j,k])}}}
N <- sum(z)
}
